# Genetic and Transcriptomic Analysis Reveal the Molecular Basis of Photoperiod-Regulated Flowering in Xishuangbanna Cucumber (*Cucumis sativus* L. var. *xishuangbannesis* Qi et Yuan)

**DOI:** 10.3390/genes12071064

**Published:** 2021-07-13

**Authors:** Zhen Tian, Molly Jahn, Xiaodong Qin, Hesbon Ochieng Obel, Fan Yang, Ji Li, Jinfeng Chen

**Affiliations:** 1 State Key Laboratory of Crop Genetics and Germplasm Enhancement, College of Horticulture, Nanjing Agricultural University, Nanjing 210095, China; 2015204016@njau.edu.cn (Z.T.); 2014204011@njau.edu.cn (X.Q.); 2019204059@njau.edu.cn (H.O.O.); 2017204028@njau.edu.cn (F.Y.); jfchen@njau.edu.cn (J.C.); 2 Jahn Research Group, USDA/FPL, Madison, WI 53726, USA; molly.jahn@jahnresearchgroup.net

**Keywords:** photoperiodic flowering process, genetic mapping, transcriptomic analysis, Xishuangbanna cucumber

## Abstract

Xishuangbanna (XIS) cucumber (*Cucumis sativus* L. var. *xishuangbannesis* Qi et Yuan), is a botanical variety of cucumber cultivars native to southwest China that possesses excellent agronomic traits for cucumber improvement. However, breeding utilization of XIS cucumber is limited due to the current poor understanding of its photoperiod-sensitive flowering characteristics. In this study, genetic and transcriptomic analysis were conducted to reveal the molecular basis of photoperiod-regulated flowering in XIS cucumber. A major-effect QTL locus *DFF1.1* was identified that controls the days to first flowering (DFF) of XIS cucumbers with a span of 1.38 Mb. Whole-genome re-sequencing data of 9 cucumber varieties with different flowering characteristics in response to photoperiod suggested that *CsaNFYA1* was the candidate gene of *DFF1.1*, which harbored a single non-synonymous mutation in its fifth exon. Transcriptomic analysis revealed the positive roles of auxin and ethylene in accelerating flowering under short-day (SD) light-dark cycles when compared with equal-day/night treatment. Carbohydrate storage and high expression levels of related genes were important reasons explaining early flowering of XIS cucumber under SD conditions. By combining with the RNA-Seq data, the co-expression network suggested that *CsaNFYA1* integrated multiple types of genes to regulate the flowering of XIS cucumber. Our findings explain the internal regulatory mechanisms of a photoperiodic flowering pathway. These findings may guide the use of photoperiod shifts to promote flowering of photoperiod-sensitive crops.

## 1. Introduction

The process of flowering is one of the most remarkable phases of plant development, and it is essential for both sexual reproduction and agricultural productivity [1]. The photoperiod is one of the main important environmental signals linked to the onset and nature of flowering among the many endogenous and exogenous elements that regulate flowering and has been extensively studied in various plant species such as *Arabidopsis*, wheat, barley and rice [2]. Flowering is accelerated by long days and delayed by short days in *Arabidopsis*, a facultative long-day (LD) plant [3]. A typical short-day (SD) plant, rice, on the other hand, shows accelerated flowering when days get shorter and delayed flowering when days get longer [4]. As a general rule, reducing plant photoperiod sensitivity is important for the global cultivation of crops to ensure improved yield regardless of day length [5]. Plant breeders desire crops with the shortest possible maturity time while preserving yield in order to avoid adverse detrimental effects triggered by climate change. This further explains why photo-insensitivity is increasingly gaining more attention in plant breeding.

The genetics and molecular biology of photoperiod-mediated flowering processes have been the subject of numerous studies. Several genes implicated in the photoperiodic flowering network have been extensively investigated. These include the florigen gene *FLOWERING LOCUS T* (*FT*) [6,7], core gene *CONSTANS* (*CO*) and Nuclear Factor Y (NF-Y) transcription factor complex [8,9], photoreceptors (such as *PHYA*, *PHYB*, *CRY1*) [10], to name a few of the most important. Previous study has treated time to flower as the target trait used to evaluate photoperiod sensitivity [11]. The flowering process consists of multiple different and important stages at which these effects might be exerted. Under the appropriate induction conditions (photoperiod, temperature and hormones, etc.), the shoot apical meristem gradually changes to the inflorescence meristem, then the meristem forms flower meristems and gradually differentiates into various flower organs, finally forming flowers [12]. Many studies have focused on the effects of photoperiod on flowering processes; however, it is still not clear which stage(s) of flowering are affected by photoperiod. Furthermore, it is necessary to examine significant changes that occurs in the most prominent points.

Cucumber, *Cucumis sativus* L. (2n = 2x = 14), is an economically significant species globally, with the fruit being consumed as a vegetable in variety of cuisines all around the world. The genetically homogeneous, inbred breeding line used in this study is called Xishuangbanna (XIS) cucumber (*Cucumis sativus* L. var. *xishuangbannesis* Qi et Yuan [13]) and is a typical SD plant. A wide range of various types of cucumber species have spread across China. Their photoperiod sensitivity varies with latitude. In China, three types of cucumbers have been developed based on photoperiod sensitivity: North China type, South China type, and Southwest China type (mainly XIS cucumber) [14]. The proposed order of photoperiod sensitivity is Southwest China type > South China type > North China type [15]. Photoperiod has no or little influence on the flowering of day-neutral North China type; however, the short-day XIS cucumber is sensitive to photoperiod changes [16]. The SD flowering character of XIS cucumber limits gene flow with other ecotypes due to asynchronous flowering. Additionally, the delay in flowering time extends the time the crop is in the field, thereby increasing risk of exposure to adverse weather before harvest that may negatively affecting crop yield. This study was undertaken to examine the photoperiodic regulation mechanisms that determine XIS cucumber flowering.

In this study, we applied SSR-based mapping and QTL-Seq analysis on the days to first flowering. RNA sequencing (RNA-Seq) analysis was conducted in the floral primordia initiation stage and the floral organ development stage to investigate the differential expression of specific genes. Finally, combined with the RNA-Seq data, we constructed the co-expression network of candidate genes that contribute to genetic variation in the photoperiod-mediated flowering pathway. Our study advances our understanding of the regulation mechanisms of photoperiodic flowering process.

## 2. Materials and Methods

### 2.1. Plant Materials and SSR-Based Mapping Analysis

CC3 (maternal line, P1) is a typical North China market type, which is insensitive to photoperiod. SWCC8 (paternal line, P2) is derived from the XIS cucumber, which is sensitive to photoperiod and requires a SD treatment for flowering in temperate regions. A population of 124 recombinant inbred lines (RILs, F_9_) from the cross of CC3 and SWCC8 [17] was used for mapping the target trait days to first flowering (DFF). The data from the RIL_9_ population were recorded in spring, 2016 (for each individual of the RIL, five plants were screened) and fall, 2016 (for each individual of the RIL, six plants were screened) in the greenhouse at Nanjing Agricultural University. DFF refers to the days from sowing to the first flower blooming (either male or female flower). Combining the previous linkage map [17] and phenotypic data of the RIL_9_ population, the QTL mapping on DFF was performed using the composite interval mapping (CIM) function of WinQTL Cartographer Version 2.5 [18,19]. The QTLs were named according to their chromosome location, trait name and the season. *Sdff1.1* and *Fdff1.1* represent the QTLs for DFF on chromosomes 1 in spring and fall detected by QTL mapping.

### 2.2. QTL-Seq Analysis

An F_2_ population from the CC3 × SWCC8 of 234 individuals was also used to further confirm the trait DFF by QTL-Seq analysis. All F_2_ plants were categorized according to the DFF. Two bulked DNA pools (early flowering pool, E-pool, 36–40 d; late flowering pool, L-pool, >52 d) were generated by mixing an equal amount of DNAs from 17 F_2_ plants in the 2017 fall using the CTAB method [20]. Thus, four Illumina libraries, two extreme bulks and parental lines CC3 and SWCC8, were sequenced individually on an Illumina HiSeq 2500 (Illumina lnc., San Diego, CA, USA). All sequencing in this study was carried out by Novogene. The raw data of QTL-Seq was uploaded successfully to NCBI Short Read Archive (SRA) under accession number SRP150560. All clean reads were aligned to the reference genome (9930 Version 2) [21]. SNP-calling was performed by SAM tools. SNP-index was assigned with the following principles: the same as CC3, SNP-index was 0; completely different, SNP-index was 1. Then the Δ (SNP-index) was calculated by subtraction the SNP-index of E-pool from that of L-pool. The average of SNP-index was calculated to reflect its distribution in a 1 Mb window size and 1 kb increment. The Δ(SNP-index) value were used to identify the candidate QTLs on DFF, 1000 permutation tests and 95% confidence level. The QTLs were named according to its trait name and chromosome location. For example, *dff1.1* refers to the QTL for DFF on chromosome 1 using QTL-Seq.

### 2.3. Prediction and Verification of Candidate Genes Regulating DFF

The QTL-Seq results and re-sequencing data of nine cucumber materials was performed to explore the possible candidate genes with nucleotide variations, located in the QTLs. Among them, CC3, CCMC and 9930 are typical North China type cucumber which are insensitive to photoperiod; Cuiyu8, L8 and Erzaozi belong to the South China type cucumber, showing a photoperiod response between the North and Southwest China type; the photoperiod-sensitive XIS cucumbers SWCC8, SWCC20 and SWCC23 were also used in this study. The re-sequencing raw data was uploaded to NCBI with the trace number SRP218666. The expression patterns of candidate genes were identified in multiple organs and different photoperiod regimes. Combined with the RNA-Seq data, the predicted co-expression network of candidate genes was presented.

### 2.4. Plant Materials and Growth Conditions of RNA-Seq Analysis

Photoperiod-sensitive XIS cucumber, SWCC8, was used for the RNA-Seq analysis [17]. Plants perceive photoperiod changes through leaves [6], therefore the transcriptome of leaves (the second true leaf below the apex) was analyzed by RNA-Seq after SD and equal-day (ED) treatments. Seeds of SWCC8 were sown into a medium (peat:vermiculite = 3:1) and grown in a chamber at 12 h/12 h (light/dark), 28 °C/18 °C (day/night), 80% relative humidity, and 800 μmol·m^−2^·s^−1^ photo flux density. Two weeks after germination, uniformly-sized seedlings with one fully unfolded true leaf were moved to two different chambers, each with a different photoperiod treatment: SD (8 h/16 h, day/night) and ED (12 h/12 h, day/night), whereas all other parameters were the same.

Leaf samples were collected from the XIS cucumber SWCC8 at varying times under SD and ED conditions (Table 1). Control samples taken at 0 days after photoperiod treatment (DAT), that is, before photoperiod treatments, were defined as initial period (IP). Based on observations of the shoot apical meristem (SAM) and later flower bud development using light microscopy of paraffin sections, samples harvested on 7 DAT, 14 DAT and 21 DAT were treated as the floral primordia initiation stage, of which the RNA was mixed to define SD1 and ED1 samples respectively. Samples collected at 37 DAT and 44 DAT were likewise pooled and considered as the floral organ development stage under SD treatment. Due to the delayed flowering, the floral organ development stage under ED treatment included as ED2 (37 DAT and 44 DAT were pooled) and ED3 (52 DAT and 59 DAT were pooled). Each sample consisted of three biological replicates collected from three individual plants. The materials were immediately placed in liquid nitrogen after harvest and stored at −80 °C.

According to the sampling time of RNA-Seq, the SAM (0 DAT, 7 DAT, 14 DAT and 21 DAT) and later flower buds (37 DAT, 44 DAT, 52 DAT and 59 DAT) were harvested, and were fixed in formalin-acetic acid-alcohol (FAA; methanol:acetic acid:50% ethyl alcohol = 5:5:90) and were then embedded, sectioned, dewaxed, and hematoxylin and eosin (HE)-staining [22]. All of the specimens were photographed under a BX41 microscope (Olympus, Tokyo, Japan).

### 2.5. RNA Extraction, Library Preparation and Sequencing

Total RNA was extracted using the Trizol Reagent (Invitrogen). Degradation and contamination of RNA were assessed on 1.5% agarose gels. RNA integrity was checked using the Nano 6000 Assay Kit of the Agilent Bioanalyzer 2100 system (Agilent Technologies, Palo Alto, California, CA, USA). High-quality RNA was pooled in equal quality following the description in the preparation of RNA-Seq materials, and 5 μg bulked RNA was used for later cDNA library preparations. Sequencing library construction was performed using the NEBNext^®^Ultra™ RNA Library Prep Kit for Illumina^®^ (NEB, Ipswich, Massachusetts, MA, USA) following the manufacturer’s protocol. The prepared libraries were sequenced on an Illumina Hiseq 2500 platform, and 150 bp paired-end reads were generated. The RNA-Seq data was deposited in NCBI under accession number SRP151164.

### 2.6. RNA-Seq Data and Enrichment Analysis

Clean reads were obtained by removing low-quality reads, adapter, and reads containing ploy-N from raw data. Then, they were mapped to the cucumber genome (http://cucurbitgenomics.org/organism/2 accessed on 1 July 2021, ver. 2i) [21] using the software TopHat (version 2.0.12) [23]. Expression levels of each gene were measured as reads per kilobase of coding sequence per million mapped reads (RPKM) [24]. DESeq R package (version 1.18.0) [25] was used to analysis differential expressed genes (DEGs) of two samples, with false discovery rate (FDR) ≤ 0.001 and |log_2_FC| ≥ 1. KEGG enrichment analysis was performed using the online website (https://www.omicshare.com/ accessed on 1 July 2021) with default instructions, and a *p*-value < 0.05 was used to determine significantly enriched KEGG entries.

### 2.7. Quantitative Real-Time PCR Analysis of RNA-Seq Data

Quantitative real-time PCR (qRT-PCR) analysis was carried out with samples described following those in RNA-Seq. Primer Premier 5.0 was used to design suitable primers (Table S1), and primers were synthesized by TSINGKE Biotech. cDNAs were reverse-transcribed using the PrimeScript™ RT reagent Kit with gDNA Eraser (Takara, Dalian, China), and qRT-PCR analysis was performed on a iCycler Real-Time PCR Detection System (Bio-Rad, Berkeley, California, CA, USA) using TaKaRa SYBR Premix Ex Taq™ (Tli RNaseH Plus, Takara, Dalian, China) with three replications. The cucumber β-actin gene (ID number: *Csa2G301530*) was used as the internal control to normalize the expression data. The relative expression of each gene was calculated using the 2^−ΔΔCT^ method [26].

### 2.8. Assay of Phenotypic and Physiological Parameters

Phenotypic data from SWCC8 plants were collected when the first flower opened under SD (8 h/16 h) and ED (12 h/12 h) treatments. The corresponding data of the days to flower, nodes and plant height when the first flower open was recorded. The assays of physiological variables were carried out by the RNA-Seq materials. Coomassie Brilliant Blue G-250 method was used for soluble protein assay [27]. The concentration of phytohormones, auxin and ethylene, were analyzed by the ELISA Kits (Sbjbio Life Science, Nanjing, China) with three biological replicates. The content of starch and sucrose were determined by the 3,5-dinitrosalicylic acid spectrophotometry [28].

### 2.9. Weighted Gene Co-Expression Network Analysis

Weighted gene co-expression network analysis (WGCNA) was performed by the RNA-Seq data (WGCNA package in R3.6.1). And genes with an averaged RPKM ≥ 5 were retained (14,492 genes) for the WGCNA analysis. The modules were obtained using the editable R program, followed with key parameters designed as: the soft threshold power was 16, and TOMType was signed. Then the gene dendrogram was used for modules identification (minModuleSize was 30). The similar modules were merged to form the final modules, with 0.25 mergeCutHeight. The module eigengene value was calculated and used to test the correlation between modules and samples. According to the topological overlap measure in each module, the associated gene pairs with corresponding edge weight value was obtained. Connectivity was defined as the sum of the edges of a node. The co-expression network was visualized using Cytoscape_v.3.7.2.

## 3. Results

### 3.1. Both Vegetative and Reproductive Growth Were Regulated by Photoperiod in XIS Cucumber

The short-day XIS cucumber SWCC8 is sensitive to photoperiod. Bo et al. [16] showed that an 8 h day/16 h night regime is the best photoperiod regime to induce early SWCC8 flowering. In our study, we designed two different light cycles, 8 h/16 h (SD) and 12 h/12 h (ED) day/night, to define the photoperiod responses of XIS cucumber SWCC8. The influence of photoperiod on flowering is exhibited as days to flower, number of nodes, plant height (Table 2). Because male flowers are first to bloom under both light cycles, we compared times until the appearance of the first male flower. With increasing lengths of daylight, the days to first flowering increased, reaching the level of significant difference. The flowering time under 12 h/12 h (98 d) was 18 days longer than that under 8 h/16 h (80 d). The nodes and plant height of first flowering also showed a similar tendency and significant difference under two light cycles. Compared with the SD regime, under ED treatment SWCC8 delays its flowering process and has more vigorous vegetative growth, accompanied by more nodes and increased plant height.

**Figure 1:**
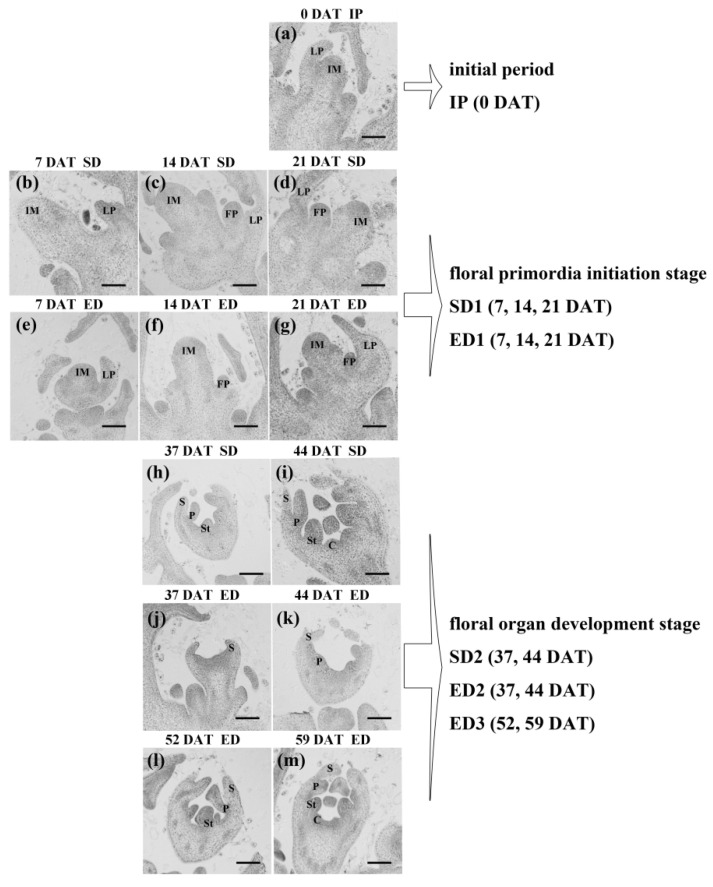
Before the first flower opens, the morphological characterizations of shoot apical meristem (SAM, 0 DAT, 7 DAT, 14 DAT, 21 DAT) and later flower bud (37 DAT, 44 DAT, 52 DAT and 59 DAT) corresponding to the time points of RNA-Seq. (**a**) The SAM before photoperiod treatments, namely initial period (IP). (**b**–**d**,**h**,**i**) The SAM and flower buds under short-day (SD, 8 h/16 h) light cycle. (**e**–**g**,**j**–**m**) The SAM and flower bud under equal-day (ED, 12 h/12 h) condition. DAT, days after photoperiod treatment; IM, inflorescence meristem; FP, floral primordia; LP, leaf primordia; S, sepal; P, petal; St, stamen; C, carpel. Bars = 100 μm.

In order to discover the effect of the photoperiod on the internal structures, paraffin sections were prepared to evaluate morphological differences in meristematic tissues throughout the flowering process. Before photoperiod treatments, during the initial period of sampling, the SAM was simple and tiny, bearing only inflorescence meristem (IM) and leaf primordia (LP) (Figure 1a). Later, the floral primordia (FP) appeared at similar times under both light cycle regimes (Figure 1c,f). The final floral organ development, however, was delayed under ED relative to SD condition. The flower buds underwent differentiation into sepals, petals, stamens, and carpels in the SD2 (Figure 1h,i), compared with only sepals and petals at ED2 (Figure 1j,k). The flower buds were differentiated into sepals, petals, stamens (Figure 1l), and later carpels (Figure 1m) until the ED3 stage under ED regime. The SD treatment accelerating the flowering process compared with ED in XIS cucumber SWCC8. In summary, photoperiod also affects the reproductive growth of XIS cucumber, and the effects of photoperiod are mainly evident at the stage of floral development where floral organs differentiate.

### 3.2. Stable QTL Loci Were Obtained on DFF by the SSR-Based Mapping

Plant photoperiod-mediated flowering is related to multiple traits. Previous studies showed that flowering time is often used as an indicator to measure plants’ photoperiod response [11,29]. In this study, North China type cucumber CC3, not sensitive to photoperiod, and Southwest China type cucumber SWCC8, sensitive to photoperiod, were used as parental lines to construct populations to predict the possible candidate loci and genes related to the target trait DFF.

In the two seasons’ field experiments, the female parent CC3 always flowered earlier than most RIL lines and male parent SWCC8; and the flowering time of F_1_ plants is close to CC3 (Figure S1a,b), suggested that the early flowering allele in CC3 might be dominant. The phenotypic statistics of DFF in the RIL_9_ population showed a continuous distribution in 2016 spring and fall, a standard normal distribution in spring (kurtosis, 0.28; skewness, 0.32) and a bimodal distribution in fall (kurtosis, −1.19; skewness, 0.01) (Table 3). The previous SSR-based genetic map [17], which contains 269 SSR markers with a total length of 705.9 cM, was employed to map the target trait DFF after combining the phenotypic data. Detailed results including detected QTLs, chromosomes, peak location, marker interval, LOD score, total phenotypic variance (R^2^), and additive effect were listed in Table 4. Only two QTLs of DFF, *Sdff1.1* and *Fdff1.1*, were identified by the SSR-based mapping; both of their phenotypic effects on variation on DFF were >10% (R^2^ = 10.6% and 21.7%, respectively), establishing these two loci as major-effect QTLs on the trait, DFF. The negative additive effect of *Sdff1.1* and *Fdff1.1* suggested that allele that comes from SWCC8 could delay the flowering time.

### 3.3. DFF1.1 Is a Major Effect QTL Controlling Photoperiod Responsive Flowering

**Figure 2:**
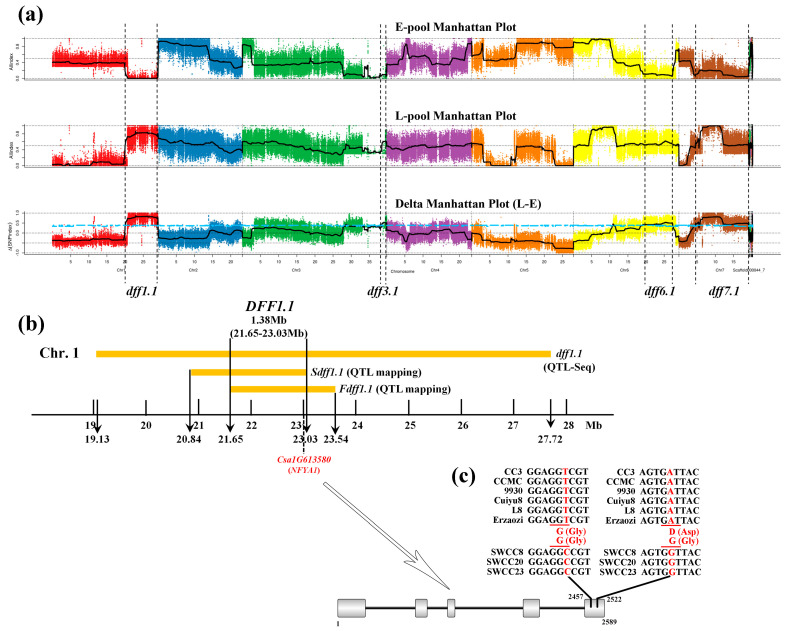
Summary of the mapping results on DFF. (**a**) The QTL-Seq results by the F_2_ population derived from the cross of CC3 and SWCC8. (**b**) The overlapping region of DFF on chromosome 1. (**c**) The structure variation of candidate gene *CsaNFYA1* (*Csa1G613580*). The only one non-synonymous mutation was detected at 2522 (D to G, Aspartate to Glycine).

The QTL loci in the SSR-based QTL mapping were then confirmed using the QTL-Seq analysis on DFF. The DFF showed a continuous distribution (Figure S1c) in the F_2_ population derived from CC3 × SWCC8 (kurtosis, −0.36; skewness, 0.58), which meets the characteristics of quantitative trait. The Illumina high-throughput sequencing resulted in 11,084,076,600 bp and 13,579,662,300 bp clean reads for the L-pool and E-pool, respectively (Table S2). The mapping rate of the four Illumina libraries was 83.59–85.45%. The comparison results were normal and can be used for subsequent variation detection analysis. By combining the information of SNP-index in E-pool and L-pool, Δ(SNP-index) was calculated and plotted against the genome positions. Finally, we identified four QTLs on DFF with 95% confidence level, namely *dff1.1*, *dff3.1*, *dff6.1* and *dff7.1* (Figure 2a).

Previous studies showed that the mapping results on flowering time were located at the similar intervals on chromosomes 1 and 6 (Table 5), when the parent lines contain cucumber varieties that are sensitive to photoperiod [17,30,31]. In this study, combined QTL mapping (*Sdff1.1* and *Fdff1.1*) and QTL-Seq (*dff1.1*, *dff3.1*, *dff6.1* and *dff7.1*) results together, three QTLs were located on chromosome 1, and all were located in a similar region with the overlapping area (*DFF1.1*) spanned 1.38 Mb (21.65–23.03 Mb) (Figure 2b). By combining with the previous studies, we suggested that there is a conservative QTL *DFF1.1* of the photoperiodic flowering time on chromosome 1.

To identify the candidate genes for the QTL, *DFF1.1*, we took the mapping results and re-sequencing data into consideration. Initially, QTL-Seq detected the large interval *dff1.1* (19.13 Mb–27.72 Mb) on chromosome 1. After selecting the most significant polymorphic SNP and Indel sites in the two bulked pools, a total of 298 nonsynonymous mutation sites in 150 nonsynonymous mutation genes (Δ(SNP-index) > 0.7) were identified (Table S3), but only 15 nonsynonymous mutation genes were left in the 1.38 Mb overlapping region *DFF1.1* (Table S4). The re-sequencing data of cucumber varieties with different photoperiod sensitivity was used for the prediction of candidate genes (Table S5). Finally, four genes, *Csa1G605740*, *Csa1G611280*, *Csa1G613550* and *Csa1G613580* (marked in red in Table S4) followed the pattern: North (CC3, CCMC) and South (Cuiyu8, L8, Erzaozi) China type cucumbers were same to the reference genome 9930, whereas all of the Southwest XIS cucumbers (SWCC8, SWCC20, SWCC23) were consistent with each other and different from the reference genome 9930. After taking the annotation information into consideration, gene *Csa1G613580* (hereafter *CsaNFYA1*), which can recognize the CCAAT-box cis-acting element in the promoter region of the *FT* gene in the photoperiodic flowering pathway [32,33], was left as a candidate for *DFF1.1*. The alignment of nucleotide sequences showed that two SNPs were detected in the exon region of *CsaNFYA1* compared with the reference genome 9930, located in the fifth exon at nucleotides 2457 (T to C) and 2522 (A to G); however, only one non-synonymous mutation was detected (D to G, Aspartate to Glycine), which came from the mutation of 2522 (A to G) (Figure 2c).

### 3.4. The Response of Hormone Was Significantly Different under SD and ED Regimes

A time-course RNA-seq analysis of leaves was performed under two kinds of photoperiod treatments. A total of 18 libraries and 56.62 (SD1-3)–60.92 (ED3-2) million raw reads were generated. After trimming and filtering, the clean reads were mapped to the cucumber genome, and the number of perfectly matched reads ranged from 22.09 (SD2-3, 40.09%) to 27.16 (IP-2, 46.84%) million (Table S6). The qRT-PCR results of six DEGs showed similar expression patterns compared with the RNA-Seq data (Figure S2), and all of the Pearson’s correlation coefficients were >0.8, further confirming the reliability of the RNA sequencing results. The correlation clustering and principal component analysis (PCA) of the 18 RNA-Seq datasets were performed (Figure S3). A close correlation between biological replicates was obtained. PCA analysis showed three distinct groups corresponding to the initial period, the floral primordia initiation stage (SD1 and ED1), and the floral organ development stage (SD2, ED2 and ED3). Even though ED2 was an extended phase under ED treatment, PCA showed that it had a very similar expression pattern to SD2 and ED3, which means that under the ED treatment, gene expression progressed to the floral organ development stage.

**Figure 3:**
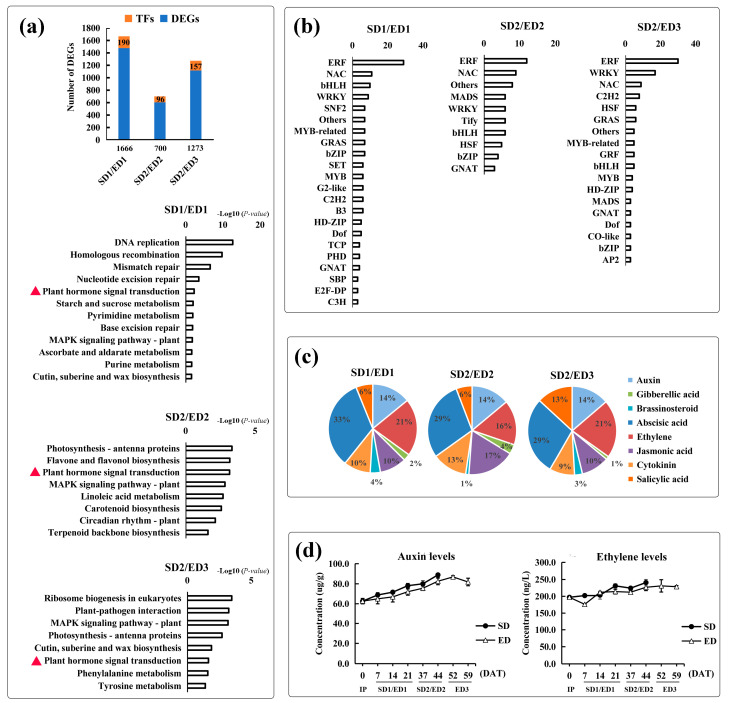
The overall analyses of DEGs in the three comparisons between SD and ED light cycles. (**a**) KEGG enrichment analysis of DEGs. All terms were listed (*p*-value < 0.05). (**b**) The detailed information of transcription factors (TFs) in the DEGs. (**c**) The pie charts represent the percentage of DEGs (including up- and down-regulated DEGs) involved in phytohormone signal transduction entry. (**d**) The concentration of auxin and ethylene according to the sampling time of RNA-Seq.

To reveal the causes of the apparent promotion of flowering process under SD when compared with the ED treatment, the significant DEGs (|log_2_FC| ≥ 1) were further analyzed in the comparisons between SD and ED photoperiod regimes, i.e., SD1/ED1, SD2/ED2 (the comparisons at the same time) and SD2/ED3 (the comparisons at the similar stage). A total of 2968 significant DEGs were produced in the three comparisons. And more DEGs were gathered in SD1/ED1 (1666) compared with SD2/ED2 (700) and SD2/ED3 (1273) (Figure 3a). Transcription factors (TFs) regulate plant growth and development through various signal transduction pathways [34]. Combined with previous studies, multiple flowering-related TFs were detected in the DEGs, such as bHLH, MADS-box, MYB and WRKY [35,36,37]. Noteworthy, ERF and NAC TFs were marked in top three in the three comparisons (Figure 3b), which used to be involved into the flowering process especially the regulation of floral organ development [38,39].

The KEGG enrichment analysis (Figure 3a) showed that entries related to replication and repair, signal transduction and material metabolism were detected in the comparison of SD1/ED1. Except signal transduction and metabolism terms, SD2/ED2 and SD2/ED3 comparisons were marked by translation and environmental adaptation entries. Notably, the “plant hormone signal transduction” entry was enriched in all three kinds of comparisons. Among them, DEGs involved in ethylene and auxin signal transduction hold a relatively high and stable proportion in the three comparisons (Figure 3c), in agreement with the pivotal roles of ethylene and auxin in the initiation of flowering and flower development [40,41,42]. The concentration of ethylene and auxin showed an upward trend under both light cycles, and their concentration under SD was almost higher than that under ED condition, especially at the floral organ development stage (Figure 3d). The higher concentration of ethylene and auxin under SD regime was consistent with the early flowering under SD, further implied the positive effects of ethylene and auxin in promoting flowering in XIS cucumber.

### 3.5. The SD Condition Accelerated the Accumulation of Carbohydrates

**Figure 4:**
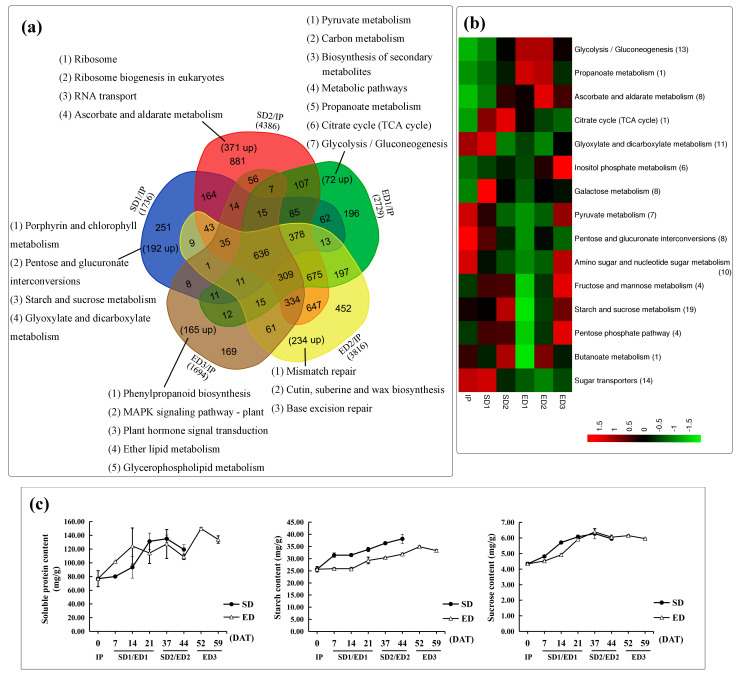
Identification of specific DEGs under SD and ED photoperiod treatments. (**a**) The Venn diagram showed the specific DEGs and its enrichment analysis (up-regulated genes) in the five comparisons (SD1/IP, SD2/IP, ED1/IP, ED2/IP and ED3/IP). (**b**) The expression patterns of carbohydrate-related DEGs in the six samples. (**c**) The content of soluble protein, starch and sucrose at the time points of RNA-Seq.

The SD photoperiod regime can promote the early XIS cucumber flowering, and the specific mechanisms and genes need further analysis. We performed Venn analysis of the five comparison pairs (SD1/IP, SD2/IP, ED1/IP, ED2/IP and ED3/IP) using the significant DEGs (|log_2_Ratio| ≥ 1). Finally, 251, 881, 196, 452 and 169 DEGs were only detected in SD1/IP, SD2/IP, ED1/IP, ED2/IP and ED3/IP, among them including 192, 371, 72, 234 and 165 up-regulated ones respectively (Figure 4a). Noteworthy, more up-regulated DEGs were enriched in SD (563) than that in ED (471). Then the further analysis was carried out by the unique up-regulated DEGs.

Although SD1 and ED1 were similar in their morphology of SAM (Figure 1c,f), DEGs were very different in SD1/IP and ED1/IP. Entries related to carbohydrates were specifically listed in SD1/IP (Pentose and glucuronate interconversions, Starch and sucrose metabolism and Glyoxylate and dicarboxylate metabolism) and ED1/IP (Pyruvate metabolism, Propanoate metabolism, Citrate cycle, Glycolysis/Gluconeogenesis), which provide nutrients for the flowering process (Figure 4a). The expression profile of carbohydrate-related DEGs were selected and presented, and finally in total of 114 DEGs were detected, including genes involved in carbohydrate biosynthesis, metabolic and transport (Figure 4b; Table S7). More DEGs began to hold a high expression level at SD1 under SD photoperiod regime; however, their high expression level were detected until ED3 stages under ED condition. Noteworthy, the number of DEGs in “Starch and sucrose metabolism” entry was the highest, so we detected the content of starch and sucrose. The measurement of starch and sucrose showed an increasing trend with the flowering process under both light cycles (Figure 4c). The sucrose content at SD1 was higher than that at ED1, which means the difference of sucrose appeared at the floral primordia initiation stage. The starch content under SD was always higher than that under ED, and was 22% higher at the highest point (14 DAT). The transport and storage of carbohydrates was delayed under ED, which was consistent with the delaying flowering under ED condition.

In SD2/IP, the KEGG enrichment analysis was obviously associated with translation process, corresponding to the higher content of soluble protein at SD2 (37 and 44 DAT) than that at ED2, including “ribosome”, “ribosome biogenesis in eukaryotes” and “RNA transport” entries (Figure 4c). As the plant structural components, a variety of proteins need to be synthesized for the formation of floral organs. Ribosomes are the cellular factories responsible for making proteins [43]. The terms related to translation process and higher content of soluble protein at SD2 were consistent with the early development of floral organs under SD than ED regime.

### 3.6. Flowering Related Genes and Transcription Factors Showed Different Expression Patterns in Response to SD and ED Photoperiod Regimes

**Figure 5:**
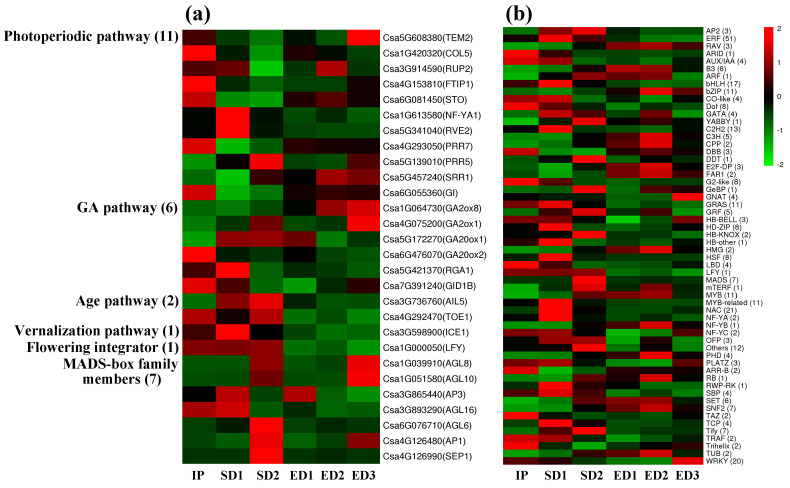
The expression profiles of DEGs related to the flowering pathways (**a**) and transcription factors (**b**).

According to a previous study in the model plant *Arabidopsis*, homologs of flowering-related genes were selected from the significant DEGs between SD and ED regimes, and their expression profiles were observed across six samples (Figure 5a; Table S8). DEGs related to multiple flowering pathways were detected, but no genes belong to the autonomous pathway. More DEGs (11) associated with the photoperiodic flowering network were detected, which further demonstrated that the photoperiodic pathway is the main flowering pathway of XIS cucumber SWCC8. It is worth noting that the flowering-promoting genes pseudo-response regulator 5 (*PRR5*) and *LEAFY* (*LFY*) have higher expression levels especially under SD condition, which is consistent with the promoting flowering of SD photoperiod regime [44,45].

The expression profiles of flowering-related TFs were compared between SD and ED conditions. Finally, 58 types and 338 TFs were detected in the significant DEGs (Figure 5b; Table S9). Because SD condition promotes the early flowering of XIS cucumber SWCC8, special attention was paid on the TFs which had high expression levels under SD regime. Among them, ERF (51), bHLH (17), C_2_H_2_ (13), CO-like (4), Dof (8), GATA (4), MYB-related (11), NAC (21) TFs had a higher expression level at SD1 stage; and AP2 (3), YABBY (1), GRF (8), MADS-box (7) TFs showed an upward regulation trend when they reached SD2 stage. As an important TFs in regulating the floral organ development [46], the expression patterns of MADS-box genes were listed in detail (Figure 5a). The expression level of MADS-box genes under SD was generally higher than that under ED, consistent with the early flowering under SD condition. For example, the expression levels of *Csa4G126990* (*SEP1*) and *Csa6G076710* (*AGL6*) were significantly higher than other genes especially at the SD2 stage. Additionally, three MADS-box transcription factors, *Csa4G126480*, *Csa1G039910*, *Csa1G051580*, exhibited an increase trend at the floral organ development stage (SD2 and ED3) under both light cycles, in agreement with the positive effects of MADS-box TFs in the flower development [46].

### 3.7. A Co-Expression Network Was Constructed by CsaNFYA1 and Related Genes

**Figure 6:**
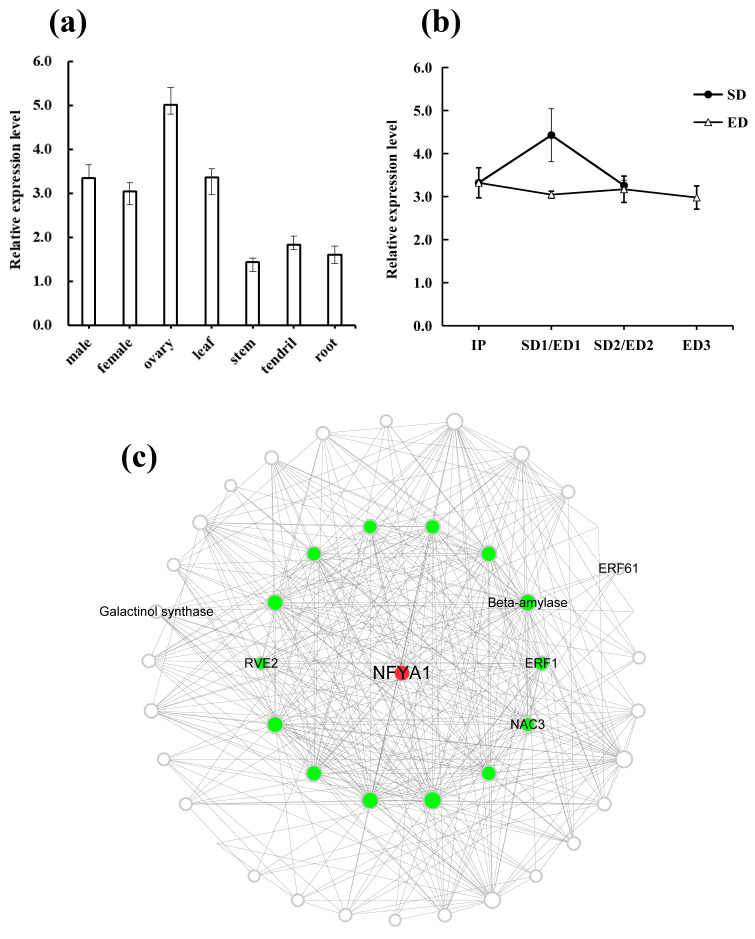
The verification of candidate gene *CsaNFYA1*. (**a**) The expression patterns of *CsaNFYA1* in multiple organs. (**b**) The expression patterns of *CsaNFYA1* under different photoperiod regimes. (**c**) The co-expression network of candidate gene *CsaNFYA1* according to the WGCNA results. *CsaNFYA1* was labeled by the red circle. Fourteen genes established the directed co-expression relationship with *CsaNFYA1* were marked by green circles. Other genes in the network were in white circles.

Analysis of the expression patterns of candidate gene *CsaNFYA1* showed that its expression has been detected in multiple organs, and the expression level in reproductive organs, male flower, female flower and ovary, was relatively higher. The expression level of *CsaNFYA1* was the highest in ovary and lowest in stem (Figure 6a). Transcriptome sequencing materials were used to determine the expression patterns of *CsaNFYA1* under different photoperiod conditions. The results showed that the expression trend of *CsaNFYA1* differed between SD and ED set-ups. The expression level of *CsaNFYA1* didn’t change much under ED condition. However, *CsaNFYA1* had a significantly high expression level at SD1 stage, the floral primordia initiation stage, which suggests that *CsaNFYA1* may promote floral transition under SD regime (Figure 6b).

Using the WGCNA results (Table S10), we constructed a network to show the co-expression relationships of *CsaNFYA1* and related genes, in which nodes represent genes and lines (edges) represent the coexpression relationships. *CsaNFYA1* was identified in the ‘mediumpurple 4’ module, which has a high correlation with SD1 stage (r = 0.93, *p*-value = 1 × 10^−8^; Figure S4). Finally, a total of 45 genes were used to construct the co-expression network and 14 (green circle) of them have a directed co-expression relationship with *CsaNFYA1* (Figure 6c). Genes associated with multiple pathways were linked to *CsaNFYA1*, such as a β-amylase gene (*Csa3G133950*), which is associated with the starch catabolism. Noteworthy, the gene *REVEILLE2* (*RVE2*, *Csa5G341040*), which regulates the photoperiod-mediated flowering time through the circadian clock pathway [47], also established a direct co-expression relationship with *CsaNFYA1*. In addition, the transcription factors *ERF1* (*Csa6G012810*) and *NAC3* (*Csa3G101810*) also had a direct co-expression relationship with *CsaNFYA1*.

## 4. Discussion

### 4.1. QTLs on Flowering Time Are Conservative in Photoperiod-Sensitive Cucumbers

Photoperiod sensitivity is a complex scientific problem, which is determined by numerous factors. A previous study on photoperiod sensitivity in maize identified 29 QTLs for plant height, leaf number, and flowering time. The three traits showed a strong correlation with each other, and they were mapped at a similar position on maize chromosomes [48]. In wheat, flowering and maturity time were mapped as a reference to measure photoperiod responses by two spring cultivars with completely different photoperiod sensitivity [49]. In addition, to reveal the genetic basis of photoperiod effect on short-day plant rice, the heading date (flowering time) was collected and analyzed [11,50]. Among them, time until flowering was used as a key indicator to study the photoperiod response [11,29]. In our study, we selected the days to first flowering to evaluate the effect of photoperiod in XIS cucumber.

Previous studies have reported the QTLs related to flowering time in cucumber (Table 5). For example, Sheng et al. [31] reported that *FT1.1* and *FT6.3* contribute to the flowering time by the cross of cucumber line Gy14 and wild cucumber line WI7221 (photoperiod sensitive). In addition, Pan et al. [30] and Bo et al. [17] identified three (*FT1.1*, *FT5.1* and *FT6.2*) and two (*fft1.1* and *fft6.1*) QTLs on flowering time, respectively by populations derived from across between semi-wild XIS cucumber (photoperiod-sensitive) and cultivated cucumber. According to the previous studies, it is worth noting that, when one of the parents is a cucumber variety that is sensitive to photoperiod, the mapping results on flowering time all contain the similar QTLs on chromosomes 1 and 6. That is, the QTLs on chromosomes 1 and 6 are two conservative QTLs related to the photoperiod-mediated flowering time. In this study, the QTL-Seq analysis found four QTLs on the days to first flowering (*dff1.1*, *dff3.1*, *dff6.1*, *dff7.1*); but the SSR-based mapping only detected the QTLs on chromosome 1 (*Sdff1.1* and *Fdff1.1*). Combined QTL-Seq, SSR-based mapping and previous studies together, it showed that there was a major-effect QTL of flowering time on chromosome 1, namely *DFF1.1* in this study (Figure 2b).

### 4.2. The Effect of Photoperiod on Flowering Process Is Different in Time

Photoperiod has many effects on plant growth and development, but perhaps the most profound involves flowering [12,51], although which floral development stages are affected, floral primordia initiation, floral organ development (differentiation into sepal, petal, stamen, gynoecia) and anthesis are controversial. It has been reported that photoperiod affects the formation of floral primordia [52]. Huang et al. [53] observed that longer day-length promoted the floral transition in *Arabidopsis*. In opium poppy, photoperiod sensitivity continues from the initiation of flower buds to later floral organ development [54]. Previous genetic mapping work has focused on flowering time in relation to photoperiod response [11,49,50]. In the present study, both vegetative and reproductive growth were regulated by different photoperiods in XIS cucumber (Table 2; Figure 1). The major influence of photoperiod was most apparent in the later floral development and at the floral organ formation (Figure 1h–m), which in turn affects the days to first flowering. The reason for this could be that the effect of photoperiod on floral primordia occurs at a certain threshold. The range of photoperiod differences in this study, 8 h/16 h and 12 h/12 h, had little effects on the initiation of floral primordia, but over time, the effects of photoperiod appeared to accumulate, evident as more pronounced effects in later development of floral organs.

### 4.3. Various Types of Genes Are Involved in the Regulation of Photoperiodic Flowering

Plant flowering is a complex process that is triggered by a combination of various internal and external cues [10]. The day-length signals stimulate the signal-receiving leaves, which stimulate the synthesis of sugars, hormones and other stimuli. Molecular genetic studies have uncovered multiple flowering-time loci and candidate genes, such as the florigen gene *FT* [55], the zinc finger transcription factor *CO* [8], a pseudo-response regulator protein *DTH7* [11]. Additionally, El-Lithy et al. [56] confirmed the close relationship between carbohydrates and flowering in *Arabidopsis* using the genetic mapping. Krizek [40] and Yamaguchi et al. [41] reported the pivotal roles of auxin during the primordia initiation and development of flower. Previous study showed that the role of ethylene signal in the flowering process is mediated through the regulation of miR156 and its target gene *SPL*, and that ethylene response factors (ERFs) act as activators during the flowering process [42,57,58].

NF-Y transcription factors, also known as heme-associated proteins (*HAPs*), are composed of three independent subunits: *NF-YA* (*HAP2*), *NF-YB* (*HAP3*), and *NF-YC* (*HAP5*) [59]. The general mechanism of NF-Y initiates with the histone-fold domain (HFD, including *NF-YB* and *NF-YC*) and *NF-YA* which form a heterotrimeric complex; the complex then binds at the CCAAT motif of target gene *FT* promoter activating its expression and initiating the flowering process [60,61,62,63,64]. *NF-YA* has the ability to identify the specific sequence of CCAAT-box in the *FT* promoter region [32]. Siriwardana et al. [33] proved that *NF-YA* mediated flowering positively in the photoperiod-dependent flowering pathway. However, Wenkel et al. [65] reported that the overexpression of *NF-YA1/4* resulted in late flowering in *Arabidopsis*. In this study, *CsaNFYA1* was treated as a candidate of the days to first flowering (Figure 2), and the expression profile showed that *CsaNFYA1* plays positive roles in the flowering transition process (Figure 6b). Plant flowering is a complex regulatory process involving multiple flowering pathways and signaling molecules [66].

## 5. Conclusions

In this study, we detected a conservative QTL locus of DFF on chromosome 1. Combined QTL-Seq and re-sequencing data showed that *CsaNFYA1* was considered as a potential candidate gene of DFF. Through the genetic and transcriptomic analysis, we found that genes specifically acting in flowering pathway, hormone and carbohydrate might cooperate with photoperiod signals to regulate XIS cucumber flowering. This paper sheds light on the specific genes that are affected by photoperiod sensitivity that affect plant flowering and provides useful information for the further study of flowering in photoperiod-sensitive plants.

## Figures and Tables

**Table 1:** The detailed experimental plan of RNA-Seq.

Photoperiod Regimes	Initial Period	Floral Primordial Initiation Stage	Floral Organ Development Stage	
SD (8 h/16 h)	IP (0 DAT)	SD1 (7, 14, 21 DAT)	SD2 (37, 44 DAT)	
ED (12 h/12 h)		ED1 (7, 14, 21 DAT)	ED2 (37, 44 DAT)	ED3 (52, 59 DAT)

DAT, days after photoperiod treatment.

**Table 2:** The detailed information of XIS cucumber, SWCC8, when the first flower opens under two photoperiod regimes.

Treatments (Day/Night)	DFF (d)	NFF	ph (cm)
SD (8 h/16 h)	80.0 ± 0.0 b	11.3 ± 2.3 b	55.5 ± 2.5 b
ED (12 h/12 h)	98.0 ± 0.0 a	16.0 ± 0.0 a	68.2 ± 2.4 a

The male flowers often to bloomed first under two light cycles, thus the male flower was used to indicate the days to first flowering in this study. The significant difference analysis was distinguished by a, b (*p* < 0.05). DFF, the days to first flowering; NFF, the nodes to first flowering; ph, the plant height to first flowering.

**Table 3:** Phenotypic variation of DFF in the RIL_9_ population at 2016 spring and fall.

Seasons	Parents		RIL Populations			
	CC3	SWCC8	Range	Mean ± SD	Kurtosis	Skewness
2016 spring	47.2 ± 0.9	87.8 ± 0.6	45.0~90.3	62.8 ± 0.7	0.28	0.32
2016 fall	32.5 ± 0.6	69.5 ± 0.9	32.0~55.0	42.5 ± 0.5	−1.19	0.01

**Table 4:** QTLs detected on DFF by the SSR-based mapping.

QTL Loci	Chr.	LOD Score	R^2^ (%)	Additive Effects	Peak Location (cM)	Marker Interval
*Sdff1.1*	1	3.5	10.6	−7.9	86.2	SSR16841-SSR23049
*Fdff1.1*	1	4.9	21.7	−3.6	88.8	SSR05723-SSR22638

**Table 5:** Summary of QTLs detected on the days to first flowering.

Number	QTL Loci	Chr.	Parental Lines (Left, Female Parent; Right, Male Parent)		References
1	*dff1.1*	1	CC3 (cultivated cucumber, insensitive)	SWCC8 (semi-wild XIS cucumber, sensitive)	QTL-Seq results in this study (from 1 to 4)
2	*dff3.1*	3			
3	*dff6.1*	6			
4	*dff7.1*	7			
5	*Sdff1.1*	1			SSR-based mapping results in this study (from 5 to 6)
6	*Fdff1.1*	1			
7	*ft1.1*	1	Gy14 (cultivated cucumber, insensitive)	WI7221 (wild cucumber, sensitive)	Sheng et al. [31] (from 7 to 10)
8	*ft1.1*	1			
9	*ft6.3*	6			
10	*ft6.3*	6			
11	*fft1.1*	1	WI7176 (semi-wild XIS cucumber, sensitive)	WI7200 (landrace originally collected from Thailand, insensitive)	Pan et al. [30] (from 11 to 15)
12	*mft1.1*	1			
13	*fft5.1*	5			
14	*fft6.2*	6			
15	*mft6.2*	6			
16	*fft1.1*	1	CC3 (cultivated cucumber, insensitive)	SWCC8 (semi-wild XIS cucumber, sensitive)	Bo et al. [17] (from 16 to 17)
17	*fft6.1*	6			

## Data Availability

Not applicable.

## Supplementary Materials

The following are available online at https://www.mdpi.com/article/10.3390/genes12071064/s1, Figure S1: Frequency distribution of the days to first flowering (DFF). Figure S2: Verify the expression profile of differential expressed genes (DEGs) in the RNA-Seq data by qRT-PCR. Figure S3: The correlation (a) and principal component analysis (b) of gene expression under SD and ED photoperiod regimes. Figure S4: The weighted gene co-expression network analysis (WGCNA) of genes in six samples of XIS cucumber. Table S1: Summary of primer sequences information. Table S2: Summary of QTL-Seq data for each sample. Table S3: Candidate genes predicted by QTL-Seq on chromosome 1. Table S4: The sequences analysis of 15 nonsynonymous mutation genes in the overlapping region (*DFF1.1*) by the resequencing data of nine cucumber varieties. Table S5: The introduction of flowering characteristics of nine cucumber varieties. Table S6: Summary of transcriptome sequencing data. Table S7: The expression patterns and annotation information of carbohydrate-related DEGs. Table S8: The expression patterns of flowering-related DEGs. Table S9: The expression levels of DEGs involved in transcription factors. Table S10: The expression level of genes participating into WGCNA.
