# Supplementary figures and images for: Genetic and Transcriptomic Analysis Reveal the Molecular Basis of Photoperiod-Regulated Flowering in Xishuangbanna Cucumber (Cucumis sativus L. var. xishuangbannesis Qi et Yuan)
Source: Genes (Basel). 2021 Jul 13;12(7):1064. doi: 10.3390/genes12071064 (PMC8304308; doi:10.3390/genes12071064)

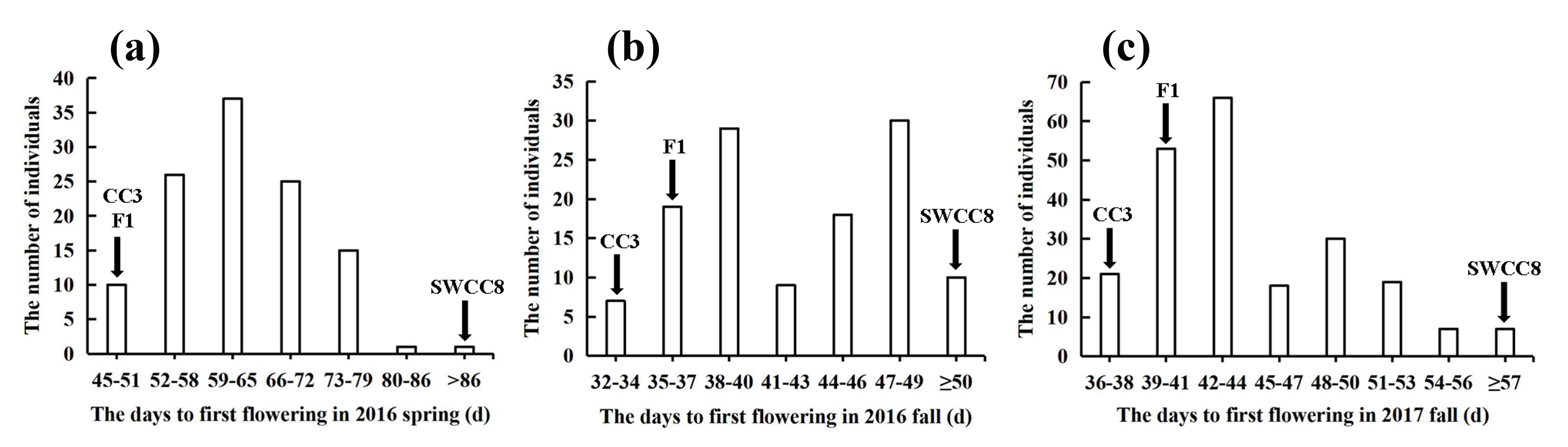

Supplement: Supplementary file 1 [file genes-12-01064-s001.zip › supplementary figures and tables/Figure S1.tif]

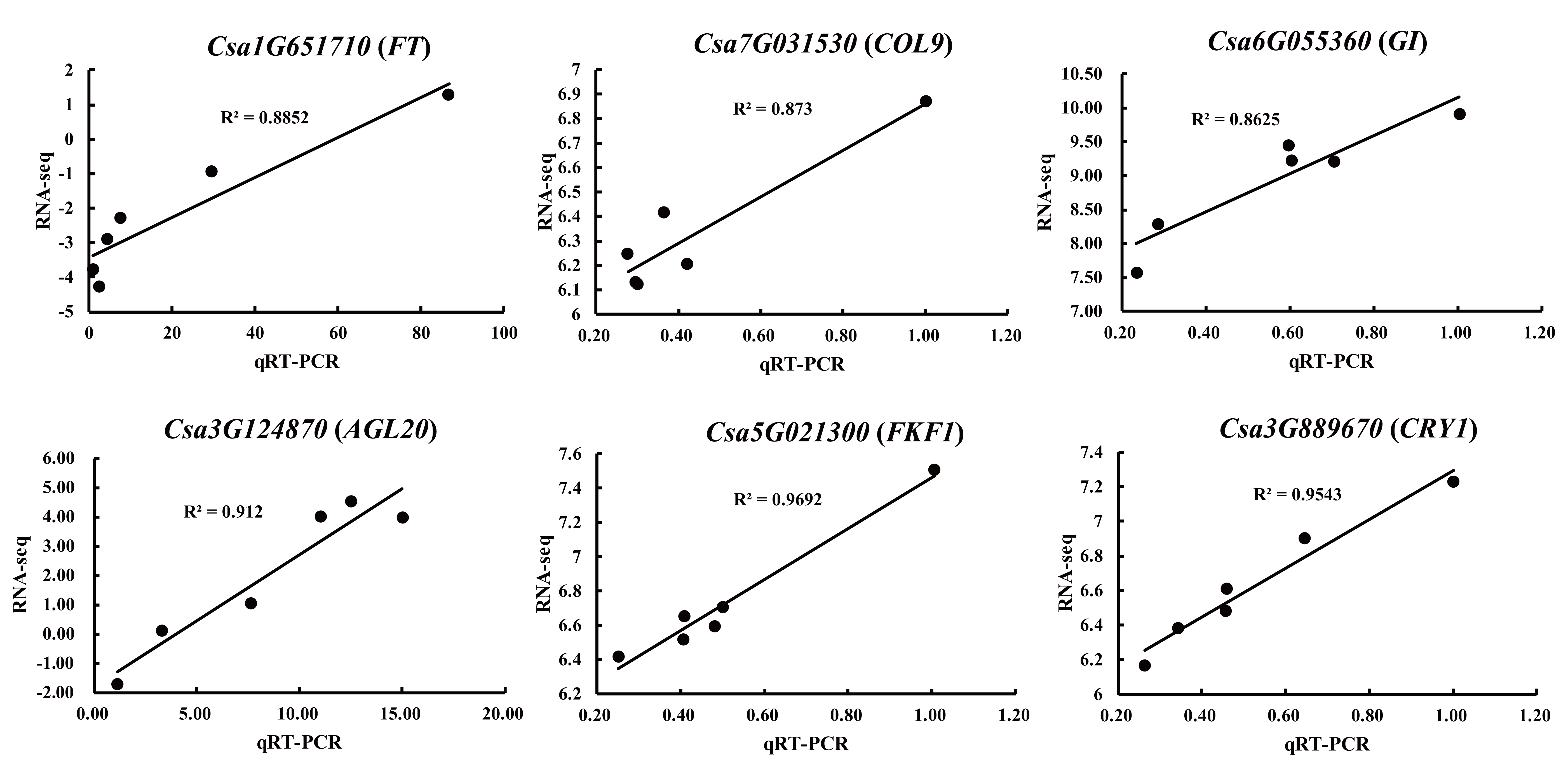

Supplement: Supplementary file 1 [file genes-12-01064-s001.zip › supplementary figures and tables/Figure S2.tif]

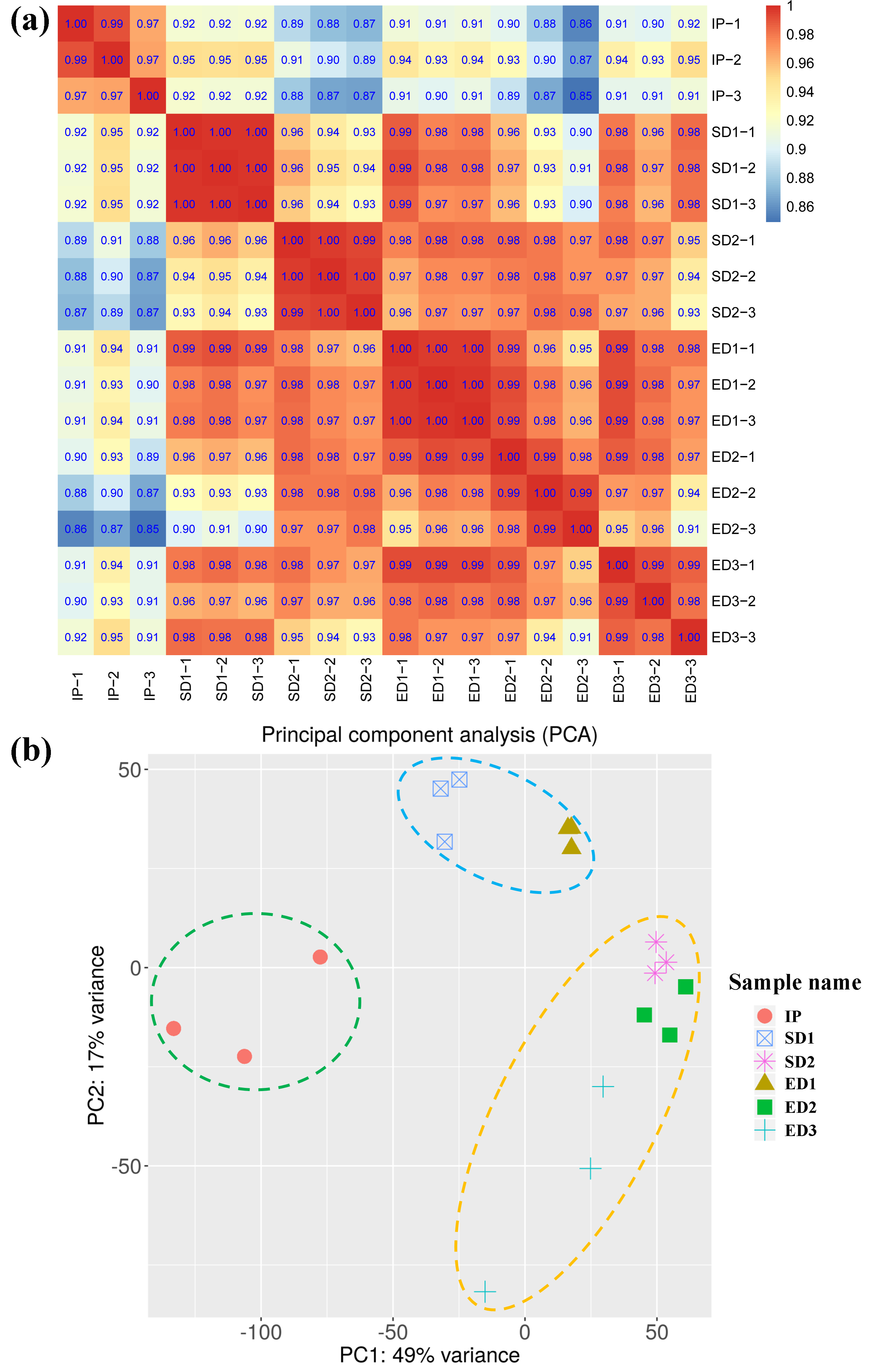

Supplement: Supplementary file 1 [file genes-12-01064-s001.zip › supplementary figures and tables/Figure S3.tif]

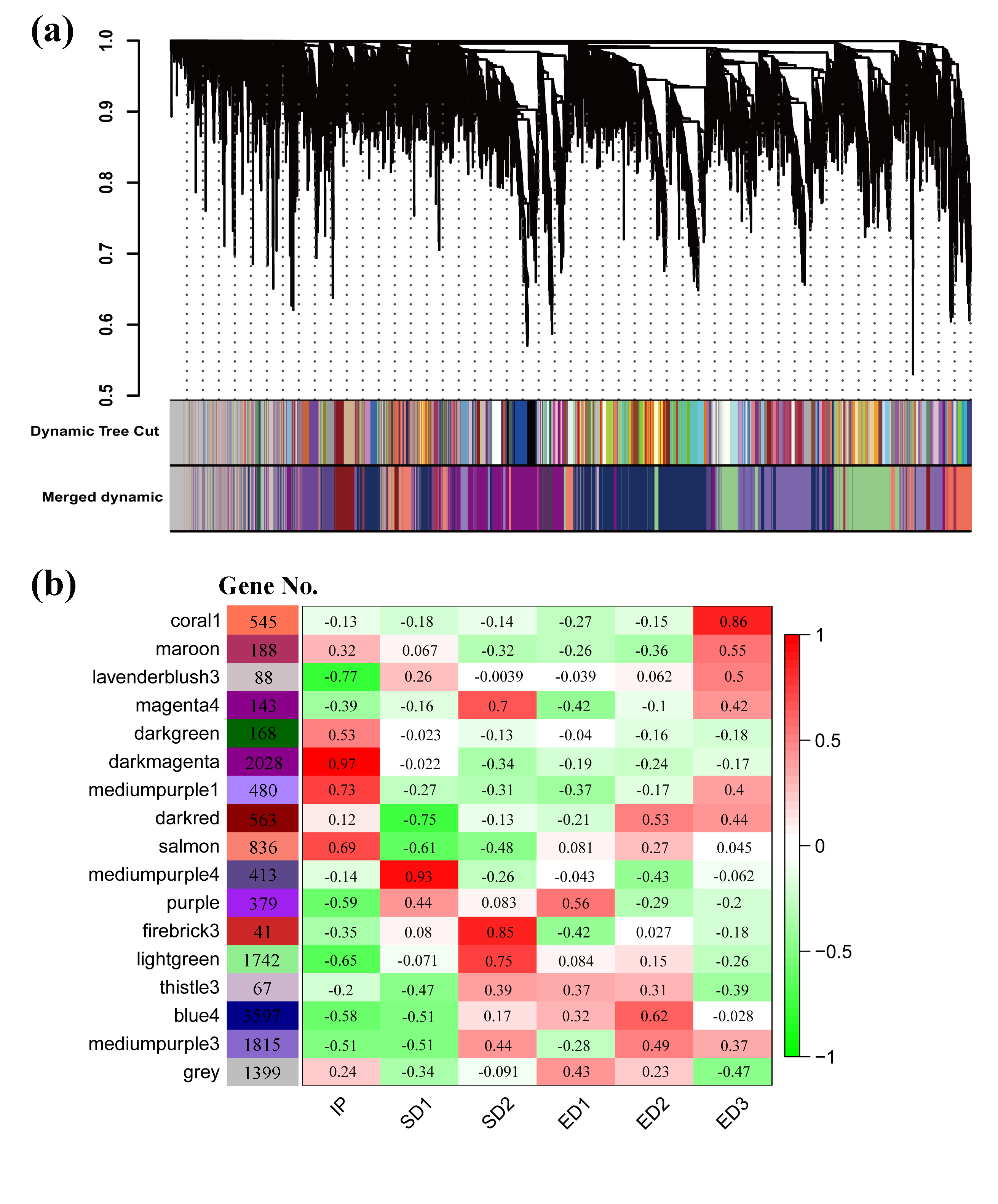

Supplement: Supplementary file 1 [file genes-12-01064-s001.zip › supplementary figures and tables/Figure S4.tif]
